# Cholest-5-en-7-one

**DOI:** 10.1107/S1600536810021598

**Published:** 2010-06-16

**Authors:** Mohd. Shaheen Khan, Othman Sulaiman, Rokiah Hashim, Ching Kheng Quah, Hoong-Kun Fun

**Affiliations:** a Bio-resource, Paper and Coatings Technology Division, School of Industrial Technology, Universiti Sains Malaysia, 11800 USM, Penang, Malaysia; b X-ray Crystallography Unit, School of Physics, Universiti Sains Malaysia, 11800 USM, Penang, Malaysia

## Abstract

In the deca­hydro­phenanthrenone ring system of the title compound, C_27_H_44_O, the two cyclo­hexane rings adopt chair conformations, whereas the cyclo­hexene ring adopts an envelope conformation. The cyclo­pentane ring is twisted. In the crystal structure, mol­ecules are stacked along the *a* axis, but no significant inter­molecular inter­actions are observed.

## Related literature

For general background to and the biological activity of steroid derivatives, see: Drach *et al.* (2000 ▶); Grover *et al.* (2007 ▶); Khan & Yusuf (2009 ▶). For the synthesis of title compound, see: Dauben & Takemura (1953 ▶); Ruiz (1958 ▶). For the stability of the temperature controller used for the data collection, see: Cosier & Glazer (1986 ▶). For details of ring conformations, see: Cremer & Pople (1975 ▶). For bond-length data, see: Allen *et al.* (1987 ▶).
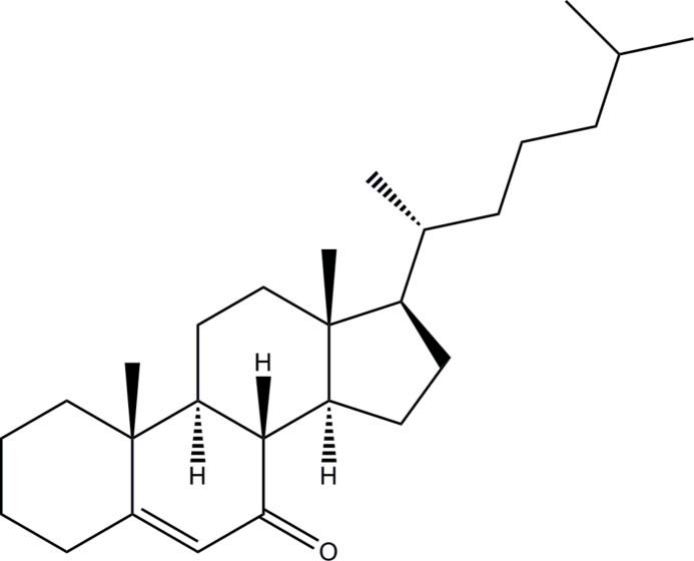


## Experimental

### 

#### Crystal data

C_27_H_44_O
*M* *_r_* = 384.62
Monoclinic, 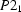

*a* = 6.3468 (13) Å
*b* = 11.517 (3) Å
*c* = 15.678 (3) Å
β = 91.470 (5)°
*V* = 1145.6 (4) Å^3^
*Z* = 2
Mo *K*α radiation
μ = 0.07 mm^−1^
*T* = 100 K
0.25 × 0.18 × 0.03 mm

#### Data collection

Bruker SMART APEXII DUO CCD area-detector diffractometer
Absorption correction: multi-scan (*SADABS*; Bruker, 2009 ▶) *T*_min_ = 0.984, *T*_max_ = 0.998
13066 measured reflections
3512 independent reflections
2776 reflections with *I* > 2σ(*I*)
*R*_int_ = 0.058

#### Refinement

*R*[*F* ^2^ > 2σ(*F* ^2^)] = 0.051
*wR*(*F* ^2^) = 0.132
*S* = 1.04
3512 reflections
252 parameters
1 restraint
H-atom parameters constrained
Δρ_max_ = 0.51 e Å^−3^
Δρ_min_ = −0.44 e Å^−3^

### 

Data collection: *APEX2* (Bruker, 2009 ▶); cell refinement: *SAINT* (Bruker, 2009 ▶); data reduction: *SAINT*; program(s) used to solve structure: *SHELXTL* (Sheldrick, 2008 ▶); program(s) used to refine structure: *SHELXTL*; molecular graphics: *SHELXTL*; software used to prepare material for publication: *SHELXTL* and *PLATON* (Spek, 2009 ▶).

## Supplementary Material

Crystal structure: contains datablocks global, I. DOI: 10.1107/S1600536810021598/is2558sup1.cif

Structure factors: contains datablocks I. DOI: 10.1107/S1600536810021598/is2558Isup2.hkl

Additional supplementary materials:  crystallographic information; 3D view; checkCIF report

## Figures and Tables

**Fig. 1.**
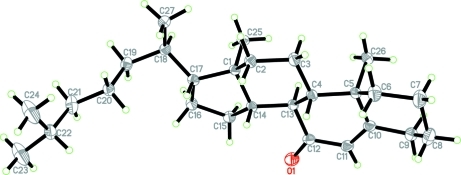
The molecular structure of the title compound, showing 50% probability displacement ellipsoids for non-H atoms and the atom-numbering scheme.

**Fig. 2.**
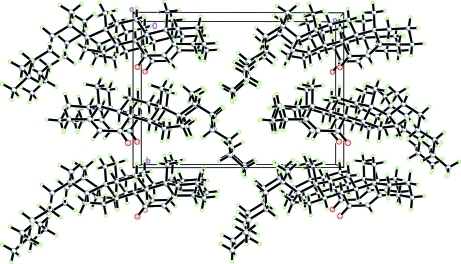
The crystal structure of the title compound viewed along the a axis.

## supplementary crystallographic information

### Comment

Steroids are compounds of biological origin and play an important role in biological systems. The dramatic expansion of steroidal chemistry came with the discovery of steroidal hormones. The discovery of several steroids with their wide application in therapy have brought about an increasing interest (Grover *et al.*, 2007). During the last decade, the major efforts of the chemists were directed towards the modification of the structures of steroids in order to enhance their biologically activity (Khan & Yusuf, 2009; Drach *et al.*, 2000).

The bond lengths (Allen *et al.*, 1987) and angles in the title compound (Fig. 1) are within normal ranges. The cyclopentane ring, C1/C14–C17 is twisted about the C1–C14 with the puckering parameters (Cremer & Pople, 1975) Q = 0.442 (3) Å and φ = 191.8 (3)°. In the tetradecahydrophenanthrene ring system, two cyclohexane rings, C5—C10 and C1—C4/C13/C14 adopt chair conformations with the puckering parameters Q = 0.539 (3) Å, Θ = 170.8 (3)° and φ = 320 (2)°; and Q = 0.585 (3) Å, Θ = 173.3 (3)° and φ = 150 (2) °, respectively, whereas C4/C5/C10—C13 adopts an envelope conformation with atom C4 deviating by 0.317 (2) Å from the mean plane through the remaining atoms, puckering parameters Q = 0.456 (3) Å, Θ = 51.6 (4)° and φ = 343.4 (4)°. The butyl (C19—C22) substituent at C18 is nearly planar, this plane lying almost perpendicular to the least-squares plane of the cyclopentane ring. The maximum deviation of the atoms C19, C20, C21 and C22 from their mean plane is 0.002 (3) Å for atoms C19, C21 and C22; and the dihedral angle between the plane of the butyl group and the least-squares plane through cyclopentane ring is 80.0 (2)°. In the crystal packing (Fig. 2), the molecules are stacked along the crystallographic *a* axis.

### Experimental

A solution of butyl chromate [*tert*-butyl alcohol (60 ml), CrO_3_ (20 g), acetic acid (84 ml) and acetic anhydride (10 ml)] (Ruiz, 1958) was added at 0 °C to a solution of cholest-5-ene (8 g) in CCl_4_ (150 ml), acetic acid (30 ml) and acetic anhydride (10 ml). The contents were refluxed for 3 h and then diluted with water. The organic layer was washed with sodium bicarbonate solution (5%) and water; and then dried over anhydrous sodium sulfate. Evaporation of the solvents under reduced pressure provided cholest-5-en-7-one which was crystallized from methanol (3.1 g), *m.p.* 128 °C (reported, *m.p.* 125–129 °C; Dauben & Takemura, 1953).

### Refinement

All H atoms were positioned geometrically and refined using a riding model, with C—H = 0.93–0.98 Å and *U*_iso_(H) = 1.2 or 1.5 *U*_eq_(C). A rotating-group model was applied for the methyl groups. The highest residual electron density peak is located at 0.07 Å from C24 and the deepest hole is located at 0.60 Å from C24. In the absence of significant anomalous dispersion, 2670 Friedel pairs were merged in the final refinement.

### Crystal data

**Table:**

C_27_H_44_O	*F*(000) = 428
*M**_r_* = 384.62	*D*_x_ = 1.115 Mg m^−^^3^
Monoclinic, *P*2_1_	Mo *K*α radiation, λ = 0.71073 Å
Hall symbol: P 2yb	Cell parameters from 2115 reflections
*a* = 6.3468 (13) Å	θ = 2.2–27.8°
*b* = 11.517 (3) Å	µ = 0.07 mm^−^^1^
*c* = 15.678 (3) Å	*T* = 100 K
β = 91.470 (5)°	Plate, colourless
*V* = 1145.6 (4) Å^3^	0.25 × 0.18 × 0.03 mm
*Z* = 2	

### Data collection

**Table:**

Bruker SMART APEXII DUO CCD area-detector diffractometer	3512 independent reflections
Radiation source: fine-focus sealed tube	2776 reflections with *I* > 2σ(*I*)
graphite	*R*_int_ = 0.058
φ and ω scans	θ_max_ = 30.2°, θ_min_ = 1.3°
Absorption correction: multi-scan (*SADABS*; Bruker, 2009)	*h* = −8→8
*T*_min_ = 0.984, *T*_max_ = 0.998	*k* = −16→16
13066 measured reflections	*l* = −21→22

### Refinement

**Table:**

Refinement on *F*^2^	Primary atom site location: structure-invariant direct methods
Least-squares matrix: full	Secondary atom site location: difference Fourier map
*R*[*F*^2^ > 2σ(*F*^2^)] = 0.051	Hydrogen site location: inferred from neighbouring sites
*wR*(*F*^2^) = 0.132	H-atom parameters constrained
*S* = 1.04	*w* = 1/[σ^2^(*F*_o_^2^) + (0.0534*P*)^2^ + 0.3776*P*] where *P* = (*F*_o_^2^ + 2*F*_c_^2^)/3
3512 reflections	(Δ/σ)_max_ = 0.001
252 parameters	Δρ_max_ = 0.51 e Å^−^^3^
1 restraint	Δρ_min_ = −0.43 e Å^−^^3^

### Special details

**Table:**

Experimental. The crystal was placed in the cold stream of an Oxford Cyrosystems Cobra open-flow nitrogen cryostat (Cosier & Glazer, 1986) operating at 100.0 (1) K.
Geometry. All e.s.d.'s (except the e.s.d. in the dihedral angle between two l.s. planes) are estimated using the full covariance matrix. The cell e.s.d.'s are taken into account individually in the estimation of e.s.d.'s in distances, angles and torsion angles; correlations between e.s.d.'s in cell parameters are only used when they are defined by crystal symmetry. An approximate (isotropic) treatment of cell e.s.d.'s is used for estimating e.s.d.'s involving l.s. planes.
Refinement. Refinement of *F*^2^ against ALL reflections. The weighted *R*-factor *wR* and goodness of fit *S* are based on *F*^2^, conventional *R*-factors *R* are based on *F*, with *F* set to zero for negative *F*^2^. The threshold expression of *F*^2^ > σ(*F*^2^) is used only for calculating *R*-factors(gt) *etc*. and is not relevant to the choice of reflections for refinement. *R*-factors based on *F*^2^ are statistically about twice as large as those based on *F*, and *R*- factors based on ALL data will be even larger.

### Fractional atomic coordinates and isotropic or equivalent isotropic displacement parameters (Å^2^)

**Table:**

	*x*	*y*	*z*	*U*_iso_*/*U*_eq_	
O1	−0.4772 (3)	0.33629 (19)	1.00165 (15)	0.0316 (5)	
C1	−0.0378 (3)	0.1067 (2)	0.87139 (16)	0.0161 (5)	
C2	0.1540 (4)	0.0845 (2)	0.92977 (16)	0.0201 (5)	
H2A	0.2407	0.1539	0.9323	0.024*	
H2B	0.2376	0.0224	0.9061	0.024*	
C3	0.0906 (4)	0.0514 (2)	1.02042 (17)	0.0225 (5)	
H3A	0.2171	0.0426	1.0559	0.027*	
H3B	0.0194	−0.0232	1.0185	0.027*	
C4	−0.0544 (4)	0.1409 (2)	1.06179 (16)	0.0185 (5)	
H4A	0.0278	0.2126	1.0678	0.022*	
C5	−0.1179 (4)	0.1052 (2)	1.15351 (16)	0.0196 (5)	
C6	0.0827 (4)	0.1099 (3)	1.21199 (18)	0.0283 (6)	
H6A	0.1757	0.0470	1.1963	0.034*	
H6B	0.1558	0.1823	1.2016	0.034*	
C7	0.0402 (5)	0.1007 (3)	1.30742 (18)	0.0344 (7)	
H7A	−0.0224	0.0259	1.3196	0.041*	
H7B	0.1719	0.1067	1.3399	0.041*	
C8	−0.1088 (5)	0.1976 (3)	1.33392 (19)	0.0382 (7)	
H8A	−0.1375	0.1906	1.3942	0.046*	
H8B	−0.0432	0.2725	1.3246	0.046*	
C9	−0.3134 (5)	0.1899 (3)	1.28214 (18)	0.0292 (6)	
H9A	−0.3867	0.1193	1.2975	0.035*	
H9B	−0.4024	0.2551	1.2965	0.035*	
C10	−0.2795 (4)	0.1900 (2)	1.18697 (17)	0.0210 (5)	
C11	−0.3927 (4)	0.2611 (2)	1.13562 (17)	0.0225 (5)	
H11A	−0.4862	0.3119	1.1610	0.027*	
C12	−0.3792 (4)	0.2643 (2)	1.04303 (17)	0.0200 (5)	
C13	−0.2455 (4)	0.1700 (2)	1.00234 (16)	0.0171 (5)	
H13A	−0.3321	0.1000	0.9958	0.021*	
C14	−0.1667 (4)	0.2043 (2)	0.91427 (16)	0.0163 (5)	
H14A	−0.0687	0.2690	0.9239	0.020*	
C15	−0.3230 (4)	0.2445 (2)	0.84445 (16)	0.0222 (5)	
H15A	−0.3637	0.3247	0.8531	0.027*	
H15B	−0.4485	0.1964	0.8432	0.027*	
C16	−0.1995 (4)	0.2306 (2)	0.76141 (17)	0.0219 (5)	
H16A	−0.1674	0.3062	0.7377	0.026*	
H16B	−0.2826	0.1874	0.7195	0.026*	
C17	0.0071 (4)	0.1643 (2)	0.78416 (16)	0.0176 (5)	
H17A	0.1178	0.2224	0.7941	0.021*	
C18	0.0752 (4)	0.0879 (2)	0.70931 (16)	0.0196 (5)	
H18A	−0.0375	0.0310	0.6992	0.024*	
C19	0.0939 (4)	0.1585 (3)	0.62657 (16)	0.0233 (5)	
H19A	0.1268	0.1053	0.5808	0.028*	
H19B	−0.0427	0.1924	0.6128	0.028*	
C20	0.2579 (4)	0.2556 (2)	0.62794 (17)	0.0221 (5)	
H20A	0.2318	0.3072	0.6754	0.027*	
H20B	0.3971	0.2223	0.6366	0.027*	
C21	0.2520 (4)	0.3250 (3)	0.54552 (19)	0.0320 (7)	
H21A	0.1114	0.3567	0.5371	0.038*	
H21B	0.2772	0.2725	0.4985	0.038*	
C22	0.4102 (5)	0.4243 (3)	0.5418 (2)	0.0320 (7)	
H22A	0.4035	0.4667	0.5958	0.038*	
C23	0.3563 (6)	0.5096 (4)	0.4707 (3)	0.0520 (7)	
H23A	0.4624	0.5689	0.4693	0.078*	
H23B	0.2219	0.5445	0.4810	0.078*	
H23C	0.3504	0.4694	0.4171	0.078*	
C24	0.6329 (5)	0.3824 (4)	0.5333 (3)	0.0520 (7)	
H24A	0.7271	0.4476	0.5350	0.078*	
H24B	0.6459	0.3422	0.4801	0.078*	
H24C	0.6680	0.3307	0.5796	0.078*	
C25	−0.1682 (4)	−0.0043 (2)	0.85817 (18)	0.0228 (5)	
H25A	−0.0796	−0.0649	0.8371	0.034*	
H25B	−0.2809	0.0104	0.8176	0.034*	
H25C	−0.2257	−0.0278	0.9115	0.034*	
C26	−0.2164 (5)	−0.0170 (2)	1.15541 (19)	0.0270 (6)	
H26A	−0.3263	−0.0224	1.1124	0.040*	
H26B	−0.2743	−0.0307	1.2105	0.040*	
H26C	−0.1099	−0.0739	1.1446	0.040*	
C27	0.2764 (4)	0.0194 (2)	0.72725 (18)	0.0234 (5)	
H27A	0.3139	−0.0225	0.6769	0.035*	
H27B	0.2542	−0.0343	0.7730	0.035*	
H27C	0.3882	0.0719	0.7432	0.035*	

### Atomic displacement parameters (Å^2^)

**Table:**

	*U*^11^	*U*^22^	*U*^33^	*U*^12^	*U*^13^	*U*^23^
O1	0.0336 (11)	0.0294 (11)	0.0322 (11)	0.0148 (9)	0.0057 (8)	0.0012 (9)
C1	0.0144 (10)	0.0140 (10)	0.0201 (12)	0.0000 (9)	0.0035 (8)	0.0013 (10)
C2	0.0156 (10)	0.0227 (12)	0.0222 (12)	0.0014 (9)	0.0048 (9)	0.0033 (11)
C3	0.0207 (12)	0.0259 (13)	0.0213 (13)	0.0073 (10)	0.0054 (10)	0.0053 (11)
C4	0.0184 (11)	0.0179 (11)	0.0192 (12)	0.0001 (9)	0.0030 (9)	0.0024 (10)
C5	0.0222 (11)	0.0170 (11)	0.0199 (12)	0.0015 (10)	0.0064 (9)	0.0022 (10)
C6	0.0264 (13)	0.0351 (15)	0.0235 (13)	0.0030 (12)	0.0012 (10)	0.0034 (13)
C7	0.0378 (16)	0.0427 (18)	0.0226 (14)	0.0050 (15)	0.0003 (12)	0.0029 (14)
C8	0.0532 (19)	0.0413 (18)	0.0203 (14)	0.0078 (16)	0.0044 (13)	−0.0032 (14)
C9	0.0379 (15)	0.0256 (13)	0.0245 (14)	0.0041 (12)	0.0090 (11)	0.0016 (12)
C10	0.0232 (12)	0.0159 (11)	0.0243 (13)	−0.0019 (10)	0.0065 (10)	−0.0012 (10)
C11	0.0229 (12)	0.0188 (12)	0.0263 (13)	0.0007 (10)	0.0079 (10)	−0.0013 (11)
C12	0.0179 (11)	0.0167 (11)	0.0255 (13)	−0.0007 (9)	0.0046 (9)	−0.0007 (10)
C13	0.0150 (10)	0.0170 (11)	0.0196 (11)	−0.0003 (9)	0.0039 (8)	0.0004 (10)
C14	0.0149 (10)	0.0144 (10)	0.0199 (12)	−0.0024 (9)	0.0037 (8)	0.0015 (10)
C15	0.0188 (11)	0.0236 (13)	0.0244 (13)	0.0078 (10)	0.0029 (9)	0.0017 (11)
C16	0.0187 (11)	0.0242 (12)	0.0229 (13)	0.0020 (10)	0.0019 (9)	0.0041 (11)
C17	0.0138 (10)	0.0163 (11)	0.0228 (12)	−0.0024 (9)	0.0025 (9)	0.0011 (10)
C18	0.0185 (11)	0.0193 (12)	0.0212 (12)	−0.0025 (9)	0.0036 (9)	−0.0020 (10)
C19	0.0213 (12)	0.0304 (14)	0.0181 (12)	0.0022 (11)	0.0003 (10)	0.0009 (11)
C20	0.0245 (12)	0.0232 (13)	0.0187 (12)	0.0020 (10)	0.0018 (9)	0.0025 (11)
C21	0.0226 (13)	0.0477 (18)	0.0258 (15)	0.0027 (13)	0.0029 (11)	0.0120 (14)
C22	0.0381 (16)	0.0290 (14)	0.0294 (16)	0.0061 (13)	0.0145 (13)	0.0078 (13)
C23	0.0340 (11)	0.0552 (16)	0.0672 (17)	0.0041 (11)	0.0088 (11)	0.0357 (15)
C24	0.0340 (11)	0.0552 (16)	0.0672 (17)	0.0041 (11)	0.0088 (11)	0.0357 (15)
C25	0.0240 (12)	0.0151 (11)	0.0297 (14)	−0.0007 (10)	0.0080 (10)	0.0009 (11)
C26	0.0386 (15)	0.0168 (12)	0.0260 (14)	−0.0028 (11)	0.0102 (12)	0.0037 (11)
C27	0.0260 (12)	0.0212 (12)	0.0232 (13)	0.0030 (10)	0.0065 (10)	0.0006 (11)

### Geometric parameters (Å, °)

**Table:**

O1—C12	1.215 (3)	C15—H15A	0.9700
C1—C2	1.526 (3)	C15—H15B	0.9700
C1—C25	1.534 (3)	C16—C17	1.551 (3)
C1—C17	1.553 (3)	C16—H16A	0.9700
C1—C14	1.554 (3)	C16—H16B	0.9700
C2—C3	1.535 (3)	C17—C18	1.537 (3)
C2—H2A	0.9700	C17—H17A	0.9800
C2—H2B	0.9700	C18—C27	1.521 (4)
C3—C4	1.537 (3)	C18—C19	1.538 (4)
C3—H3A	0.9700	C18—H18A	0.9800
C3—H3B	0.9700	C19—C20	1.528 (4)
C4—C13	1.547 (3)	C19—H19A	0.9700
C4—C5	1.558 (3)	C19—H19B	0.9700
C4—H4A	0.9800	C20—C21	1.519 (4)
C5—C10	1.519 (3)	C20—H20A	0.9700
C5—C26	1.541 (4)	C20—H20B	0.9700
C5—C6	1.550 (4)	C21—C22	1.524 (5)
C6—C7	1.531 (4)	C21—H21A	0.9700
C6—H6A	0.9700	C21—H21B	0.9700
C6—H6B	0.9700	C22—C24	1.502 (4)
C7—C8	1.527 (5)	C22—C23	1.518 (5)
C7—H7A	0.9700	C22—H22A	0.9800
C7—H7B	0.9700	C23—H23A	0.9600
C8—C9	1.516 (5)	C23—H23B	0.9600
C8—H8A	0.9700	C23—H23C	0.9600
C8—H8B	0.9700	C24—H24A	0.9600
C9—C10	1.513 (4)	C24—H24B	0.9600
C9—H9A	0.9700	C24—H24C	0.9600
C9—H9B	0.9700	C25—H25A	0.9600
C10—C11	1.344 (4)	C25—H25B	0.9600
C11—C12	1.457 (4)	C25—H25C	0.9600
C11—H11A	0.9300	C26—H26A	0.9600
C12—C13	1.528 (3)	C26—H26B	0.9600
C13—C14	1.532 (3)	C26—H26C	0.9600
C13—H13A	0.9800	C27—H27A	0.9600
C14—C15	1.530 (4)	C27—H27B	0.9600
C14—H14A	0.9800	C27—H27C	0.9600
C15—C16	1.545 (3)		
			
C2—C1—C25	111.2 (2)	C16—C15—H15A	111.0
C2—C1—C17	115.98 (18)	C14—C15—H15B	111.0
C25—C1—C17	110.3 (2)	C16—C15—H15B	111.0
C2—C1—C14	106.4 (2)	H15A—C15—H15B	109.0
C25—C1—C14	111.92 (18)	C15—C16—C17	107.5 (2)
C17—C1—C14	100.51 (18)	C15—C16—H16A	110.2
C1—C2—C3	111.90 (19)	C17—C16—H16A	110.2
C1—C2—H2A	109.2	C15—C16—H16B	110.2
C3—C2—H2A	109.2	C17—C16—H16B	110.2
C1—C2—H2B	109.2	H16A—C16—H16B	108.5
C3—C2—H2B	109.2	C18—C17—C16	111.0 (2)
H2A—C2—H2B	107.9	C18—C17—C1	119.3 (2)
C2—C3—C4	113.4 (2)	C16—C17—C1	103.87 (18)
C2—C3—H3A	108.9	C18—C17—H17A	107.4
C4—C3—H3A	108.9	C16—C17—H17A	107.4
C2—C3—H3B	108.9	C1—C17—H17A	107.4
C4—C3—H3B	108.9	C27—C18—C17	114.0 (2)
H3A—C3—H3B	107.7	C27—C18—C19	110.3 (2)
C3—C4—C13	111.1 (2)	C17—C18—C19	111.7 (2)
C3—C4—C5	112.6 (2)	C27—C18—H18A	106.8
C13—C4—C5	113.32 (19)	C17—C18—H18A	106.8
C3—C4—H4A	106.4	C19—C18—H18A	106.8
C13—C4—H4A	106.4	C20—C19—C18	116.3 (2)
C5—C4—H4A	106.4	C20—C19—H19A	108.2
C10—C5—C26	107.6 (2)	C18—C19—H19A	108.2
C10—C5—C6	109.0 (2)	C20—C19—H19B	108.2
C26—C5—C6	110.3 (2)	C18—C19—H19B	108.2
C10—C5—C4	110.0 (2)	H19A—C19—H19B	107.4
C26—C5—C4	111.9 (2)	C21—C20—C19	111.8 (2)
C6—C5—C4	108.0 (2)	C21—C20—H20A	109.3
C7—C6—C5	114.4 (2)	C19—C20—H20A	109.3
C7—C6—H6A	108.7	C21—C20—H20B	109.3
C5—C6—H6A	108.7	C19—C20—H20B	109.3
C7—C6—H6B	108.7	H20A—C20—H20B	107.9
C5—C6—H6B	108.7	C20—C21—C22	115.2 (3)
H6A—C6—H6B	107.6	C20—C21—H21A	108.5
C8—C7—C6	109.9 (3)	C22—C21—H21A	108.5
C8—C7—H7A	109.7	C20—C21—H21B	108.5
C6—C7—H7A	109.7	C22—C21—H21B	108.5
C8—C7—H7B	109.7	H21A—C21—H21B	107.5
C6—C7—H7B	109.7	C24—C22—C23	109.8 (3)
H7A—C7—H7B	108.2	C24—C22—C21	112.6 (3)
C9—C8—C7	109.8 (3)	C23—C22—C21	112.2 (3)
C9—C8—H8A	109.7	C24—C22—H22A	107.3
C7—C8—H8A	109.7	C23—C22—H22A	107.3
C9—C8—H8B	109.7	C21—C22—H22A	107.3
C7—C8—H8B	109.7	C22—C23—H23A	109.5
H8A—C8—H8B	108.2	C22—C23—H23B	109.5
C10—C9—C8	112.7 (2)	H23A—C23—H23B	109.5
C10—C9—H9A	109.0	C22—C23—H23C	109.5
C8—C9—H9A	109.0	H23A—C23—H23C	109.5
C10—C9—H9B	109.0	H23B—C23—H23C	109.5
C8—C9—H9B	109.0	C22—C24—H24A	109.5
H9A—C9—H9B	107.8	C22—C24—H24B	109.5
C11—C10—C9	120.3 (2)	H24A—C24—H24B	109.5
C11—C10—C5	122.7 (2)	C22—C24—H24C	109.5
C9—C10—C5	117.0 (2)	H24A—C24—H24C	109.5
C10—C11—C12	124.6 (2)	H24B—C24—H24C	109.5
C10—C11—H11A	117.7	C1—C25—H25A	109.5
C12—C11—H11A	117.7	C1—C25—H25B	109.5
O1—C12—C11	120.5 (2)	H25A—C25—H25B	109.5
O1—C12—C13	123.0 (2)	C1—C25—H25C	109.5
C11—C12—C13	116.5 (2)	H25A—C25—H25C	109.5
C12—C13—C14	113.0 (2)	H25B—C25—H25C	109.5
C12—C13—C4	109.7 (2)	C5—C26—H26A	109.5
C14—C13—C4	109.24 (18)	C5—C26—H26B	109.5
C12—C13—H13A	108.3	H26A—C26—H26B	109.5
C14—C13—H13A	108.3	C5—C26—H26C	109.5
C4—C13—H13A	108.3	H26A—C26—H26C	109.5
C15—C14—C13	120.13 (19)	H26B—C26—H26C	109.5
C15—C14—C1	104.36 (19)	C18—C27—H27A	109.5
C13—C14—C1	113.01 (19)	C18—C27—H27B	109.5
C15—C14—H14A	106.1	H27A—C27—H27B	109.5
C13—C14—H14A	106.1	C18—C27—H27C	109.5
C1—C14—H14A	106.1	H27A—C27—H27C	109.5
C14—C15—C16	103.77 (19)	H27B—C27—H27C	109.5
C14—C15—H15A	111.0		
			
C25—C1—C2—C3	−64.6 (3)	C5—C4—C13—C12	55.4 (3)
C17—C1—C2—C3	168.2 (2)	C3—C4—C13—C14	−52.3 (3)
C14—C1—C2—C3	57.4 (3)	C5—C4—C13—C14	179.8 (2)
C1—C2—C3—C4	−55.3 (3)	C12—C13—C14—C15	−53.9 (3)
C2—C3—C4—C13	51.4 (3)	C4—C13—C14—C15	−176.4 (2)
C2—C3—C4—C5	179.7 (2)	C12—C13—C14—C1	−177.8 (2)
C3—C4—C5—C10	−173.8 (2)	C4—C13—C14—C1	59.7 (3)
C13—C4—C5—C10	−46.6 (3)	C2—C1—C14—C15	166.22 (19)
C3—C4—C5—C26	−54.2 (3)	C25—C1—C14—C15	−72.2 (2)
C13—C4—C5—C26	72.9 (3)	C17—C1—C14—C15	44.9 (2)
C3—C4—C5—C6	67.4 (3)	C2—C1—C14—C13	−61.6 (2)
C13—C4—C5—C6	−165.5 (2)	C25—C1—C14—C13	60.0 (3)
C10—C5—C6—C7	49.0 (3)	C17—C1—C14—C13	177.14 (19)
C26—C5—C6—C7	−68.9 (3)	C13—C14—C15—C16	−161.7 (2)
C4—C5—C6—C7	168.5 (3)	C1—C14—C15—C16	−33.7 (2)
C5—C6—C7—C8	−57.5 (4)	C14—C15—C16—C17	9.3 (3)
C6—C7—C8—C9	58.3 (3)	C15—C16—C17—C18	147.7 (2)
C7—C8—C9—C10	−54.5 (3)	C15—C16—C17—C1	18.4 (3)
C8—C9—C10—C11	−131.4 (3)	C2—C1—C17—C18	83.6 (3)
C8—C9—C10—C5	49.8 (3)	C25—C1—C17—C18	−43.9 (3)
C26—C5—C10—C11	−104.0 (3)	C14—C1—C17—C18	−162.2 (2)
C6—C5—C10—C11	136.3 (3)	C2—C1—C17—C16	−152.2 (2)
C4—C5—C10—C11	18.1 (3)	C25—C1—C17—C16	80.2 (2)
C26—C5—C10—C9	74.6 (3)	C14—C1—C17—C16	−38.0 (2)
C6—C5—C10—C9	−45.0 (3)	C16—C17—C18—C27	−179.6 (2)
C4—C5—C10—C9	−163.2 (2)	C1—C17—C18—C27	−59.0 (3)
C9—C10—C11—C12	−177.1 (3)	C16—C17—C18—C19	54.5 (3)
C5—C10—C11—C12	1.5 (4)	C1—C17—C18—C19	175.1 (2)
C10—C11—C12—O1	−175.3 (3)	C27—C18—C19—C20	−65.8 (3)
C10—C11—C12—C13	7.4 (4)	C17—C18—C19—C20	62.2 (3)
O1—C12—C13—C14	25.7 (3)	C18—C19—C20—C21	−175.9 (2)
C11—C12—C13—C14	−157.1 (2)	C19—C20—C21—C22	179.7 (2)
O1—C12—C13—C4	147.9 (2)	C20—C21—C22—C24	71.8 (4)
C11—C12—C13—C4	−34.9 (3)	C20—C21—C22—C23	−163.7 (3)
C3—C4—C13—C12	−176.7 (2)		
